# A VPS33A-binding motif on syntaxin 17 controls autophagy completion in mammalian cells

**DOI:** 10.1074/jbc.RA118.005947

**Published:** 2019-01-17

**Authors:** Rebecca S. Saleeb, Deirdre M. Kavanagh, Alison R. Dun, Paul A. Dalgarno, Rory R. Duncan

**Affiliations:** From the Edinburgh Super-Resolution Imaging Consortium, Institute of Biological Chemistry, Biophysics, and Bioengineering, School of Engineering and Physical Sciences, Heriot-Watt University, Edinburgh EH14 4AS, United Kingdom

**Keywords:** autophagy, SNARE proteins, imaging, fluorescence resonance energy transfer (FRET), membrane, syntaxin 17, FLIM-FRET, membrane trafficking, protein degradation, lysosome, phagosome, VPS33A

## Abstract

Autophagy is an intracellular degradation pathway that transports cytoplasmic material to the lysosome for hydrolysis. It is completed by SNARE-mediated fusion of the autophagosome and endolysosome membranes. This process must be carefully regulated to maintain the organization of the membrane system and prevent mistargeted degradation. As yet, models of autophagosomal fusion have not been verified within a cellular context because of difficulties with assessing protein interactions *in situ*. Here, we used high-resolution fluorescence lifetime imaging (FLIM)-FRET of HeLa cells to identify protein interactions within the spatiotemporal framework of the cell. We show that autophagosomal syntaxin 17 (Stx17) heterotrimerizes with synaptosome-associated protein 29 (SNAP29) and vesicle-associated membrane protein 7 (VAMP7) *in situ*, highlighting a functional role for VAMP7 in autophagosome clearance that has previously been sidelined in favor of a role for VAMP8. Additionally, we identified multimodal regulation of SNARE assembly by the Sec1/Munc18 (SM) protein VPS33A, mirroring other syntaxin–SM interactions and therefore suggesting a unified model of SM regulation. Contrary to current theoretical models, we found that the Stx17 N-peptide appears to interact in a positionally conserved, but mechanistically divergent manner with VPS33A, providing a late “go, no-go” step for autophagic fusion via a phosphoserine master-switch. Our findings suggest that Stx17 fusion competency is regulated by a phosphosite in its N-peptide, representing a previously unknown regulatory step in mammalian autophagy.

## Introduction

Macroautophagy (henceforth autophagy) is a bulk intracellular degradation pathway that transports cytoplasmic material to the lysosome for hydrolysis. Autophagy has an important role in protein turnover, the elimination of cytotoxic material, and energy homeostasis. The wide range of conditions linked to autophagy (1) highlight its importance in health and disease.

Autophagy sequesters cargo by the growth of an open-ended double-membrane vesicle, named a phagophore, which forms *de novo* (2) upon nucleation at endoplasmic reticulum–mitochondrial contact sites (3). Closure of this structure forms an autophagosome, which travels to the endolysosome and deposits its contents by membrane fusion. Isolation of cargo by sequestration rather than vesicle budding is a seemingly unique mode of membrane trafficking.

SNARE^4^ proteins are established molecular drivers of membrane fusion (4). A subset of these proteins form a fusion event–specific four-α-helical complex, composed of a Qa-, Qb-, Qc-, and R-SNARE motif (5), which spans the apposing membranes. Amalgamation of the lipid bilayers is promoted by “zippering” of the SNARE complex from the N to the C terminus (6). The Qa-SNARE, Stx17, has been identified on the autophagosome (7), suggesting that autophagy completion is a SNARE-mediated process. Mammalian Stx17 has been shown to associate *in vitro* with the soluble Qbc-SNARE, SNAP29, and the endolysosomal R-SNARE, VAMP8 (7).

Organization of any membrane system depends on careful regulation of fusion specificity, for which combinatorial SNARE complex formation alone is not sufficient (8). Regulation may be accomplished by Sec1/Munc18 (SM) proteins, which have been observed to have multiple regulatory mechanisms, including both the promotion and inhibition of fusion, dependent on the SM-SNARE pair and their binding mode (9). Fusion promotion is accomplished by stabilization of the SNARE bundle (10, 11), the enhancement of complex fusogenicity (12), or the recruitment of SNARE proteins to the fusion site (13), with inhibition mediated by stabilization of a “closed” conformation of syntaxin (14, 15).

The SM protein VPS33A reportedly promotes both SNARE-mediated autophagosome clearance (16) and late endosome–lysosome fusion (17). VPS33A is unique among mammalian SM proteins in forming an integral part of multisubunit tethering complexes, one of which is the late endosomal homotypic fusion and vacuole protein sorting (HOPS) complex (18), from which it likely modulates autophagosome clearance (19). Importantly, VPS33A is also divergent in being considered an SM protein that cannot interact with a cognate syntaxin N-peptide region, because it lacks an acceptor binding pocket thought to be essential for such an interaction mode (20). As such, VPS33A is currently believed to have a promotion-only role in regulation.

In the present study, we aimed to confirm *in situ* the mammalian SNARE machinery involved in final-stage autophagy and to determine how this fusion event is controlled at a molecular level. Such rigorous *in situ* analysis of the autophagosomal SNARE fusion model is still needed to validate *in vitro* findings. Indeed, the SNARE composition remains under question (21, 22).

We query in particular the accepted role of VAMP8 in the autophagosomal SNARE complex. In contrast to current thinking about mammalian autophagy, the R-SNARE governing autophagosome fusion in *Drosophila melanogaster* is VAMP7 (23). This discrepancy is broadly accepted because *D. melanogaster* has no equivalent to VAMP8; VAMP7, which is 62% similar, is considered its closest homolog. However, VAMP8 lacks the regulatory longin domain encoded by *D. melanogaster* VAMP7, the *Saccharomyces cerevisiae* autophagosomal R-SNARE, Ykt6, and mammalian VAMP7, which can also be immunoprecipitated with Stx17 (7). This anomaly for mammalian VAMP8 highlights it as a curious candidate SNARE for autophagy in mammals. Furthermore, homotypic and heterotypic endosomal fusion events appear to be differentially mediated by VAMP8 and VAMP7 (24), respectively, placing VAMP7 as an arguably more credible candidate for the heterotypic fusion of the autophagosome and endolysosome.

Protein interactions are difficult to study *in situ*, and fluorescence colocalization is often used to confirm *in vitro* interactions, yet diffraction-limited microscopy is restricted to ∼250 nm resolution (25), from which molecular association cannot be concluded. This distinction is particularly relevant to studies of autophagosome clearance; the convergence of autophagy with other trafficking pathways could lead to the coincidental accumulation of SNAREs on the lysosomal membrane, whereas the binding promiscuity of SNARE proteins (26) may affect *in vitro* data.

To address this, autophagosomal SNARE interactions were studied *in situ* using fluorescence lifetime imaging microscopy (FLIM) to detect FRET between fluorophores in less than 8-nm proximity (27). With this technique, we have investigated the regulation and formation of the autophagosomal SNARE complex in HeLa cells. Our data show that contrary to current models, VAMP7 associates significantly with Stx17 and correlates with autophagosomal compartments in a fusion-dependent manner. Additionally, the clear punctate pattern of the binary SNARE heterodimer highlights the necessity of an inhibitory regulator to prevent premature formation of this complex, which may result in early or ectopic fusion events. The data we present here suggest that Stx17 fusion competency is regulated by an N-peptide phosphosite, presenting a hitherto unidentified regulatory step in autophagy. We show that its phosphorylation status mediates a novel second mode interaction of VPS33A, contradicting the accepted classification of VPS33A as a noninhibitory SM protein (20).

## Results

### Stx17-resident autophagosomes co-occur with SNAP29 and VAMP7

**Figure 1.**
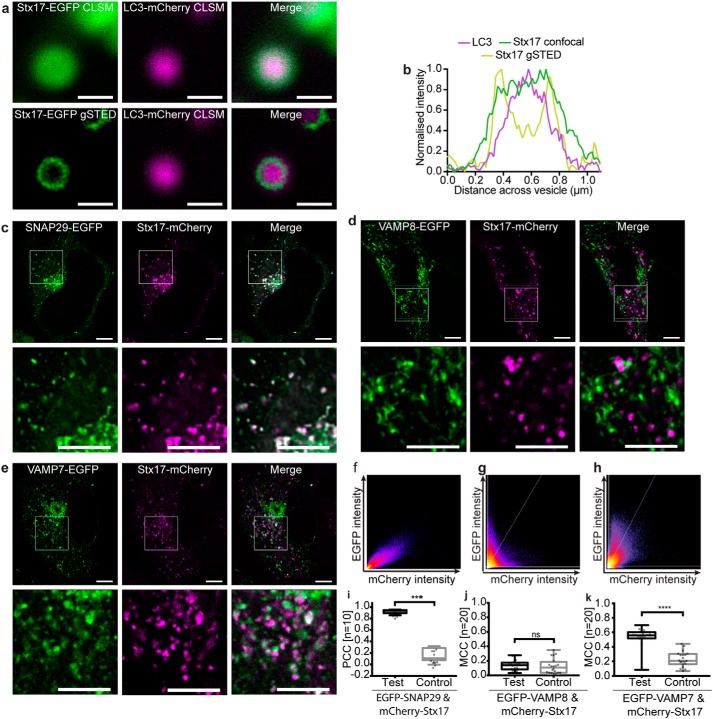
**Autophagosomal Stx17 colocalizes significantly with SNAP29 and VAMP7.** *a*, representative confocal and gSTED images of membrane-bound Stx17 rings (*left*) surrounding LC3-labeled autophagosomes (*center*) and a merge of these images (*right*) from rapamycin-treated HeLa cells. *Scale bars*, 500 nm. *b*, intensity line profile through the punctum in *a*, demonstrating membrane localization of Stx17 resolved by gSTED. *c–e*, single-channel and merged images of rapamycin-treated HeLa cells expressing Stx17 with either SNAP29 (*c*), VAMP8 (*d*), or VAMP7 (*e*) and a merge of both channels. *Scale bars*, 10 μm for full field and 3 μm for *zoomed regions. f–h*, frequency scatter plots of reported single-pixel intensity values in each channel presented in *c–e. i*, quantification of SNAP29 and Stx17 colocalization using Pearson's correlation coefficient (*PCC*) demonstrating significantly higher correlation than in negative controls generated by single-channel 90° rotation. *j* and *k*, quantification of Stx17 puncta colocal with VAMP8 (*j*) or VAMP7 (*k*) puncta using Manders correlation coefficient. All box-and-whisker plots represent the median (*central line*), 25th and 75th quartile (*box*), and the minimum and maximum value (*whiskers*). *ns*, nonsignificant (*p* ≥ 0.05); ***, *p* = 0.0001–0.0005; ****, *p* < 0.0001.

Stx17 is well established as the autophagosomal SNARE (7), redistributing to punctate structures colocal with the autophagosome marker, LC3 (28), upon the induction of autophagy. We confirmed this finding (Fig. S1, *a–c*) in our experimental model, which uses fluorophore-tagged protein exogenously expressed in HeLa cells treated with rapamycin to induce autophagy. To first verify that exogenously expressed Stx17 is properly localized and fully active, we used gated stimulated emission depletion (gSTED) microscopy to superresolve Stx17 localization and fusion inhibition studies to assess normal autophagic flux. As expected for a correctly folded integral membrane protein, EGFP-Stx17 is restricted to LC3-resident ring structures when superresolved (Fig. 1, *a* and *b*). Additionally, the correlation between mCherry-Stx17 and EGFP-LC3, the latter of which alone is quenched by the lysosomal environment (29), increases when fusion is chemically blocked with bafilomycin A_1_ (Fig. S1, *d* and *e*), suggesting that these are indeed fusion-competent autophagosomes.

Given the known size of autophagosomes, ranging from of 0.5–1.5 μm (30), diffraction-limited confocal fluorescence microscopy was used to confirm the expected signal correlation between SNAREs on vesicular structures during autophagy. Stx17 and SNAP29 demonstrate a strong signal correlation that follows a linear relationship and significantly co-varies as determined by Pearson's analysis (Fig. 1, *c*, *f*, and *i*). Perhaps surprisingly, however, poor signal correlation is evident with VAMP8, the favored autophagosomal R-SNARE (Fig. 1, *d*, *g*, and *j*). An alternative endolysosome-resident R-SNARE, VAMP7, instead demonstrated partial correlation with Stx17 (Fig. 1, *e*, *h*, and *k*), as would be expected if their co-targeting is restricted to post-fusion compartments. The nearly total correlation of Stx17 with SNAP29 but partial correlation with VAMP7 hints at a multistep assembly process analogous to the regulated exocytotic complex (31), where the Q-SNARE binary heterodimer first forms, incorporating the R-SNARE in a short-lived *trans*-SNARE complex that spans apposing membranes, becoming a *cis*-complex following fusion. The colocalization of such subpopulations is poorly suited to metrics of co-variance (Fig. S2), and instead the fraction of signal co-distribution was ascertained by the Manders correlation coefficient (32), showing significant correlation of Stx17 with VAMP7, but not VAMP8 (Fig. 1, *j* and *k*). Importantly, unlike VAMP8, we find that VAMP7 also correlates with LC3 in a fusion-dependent manner (Fig. S3).

### SNAP29 and VAMP7 interact with Stx17 via its SNARE domain

**Figure 2.**
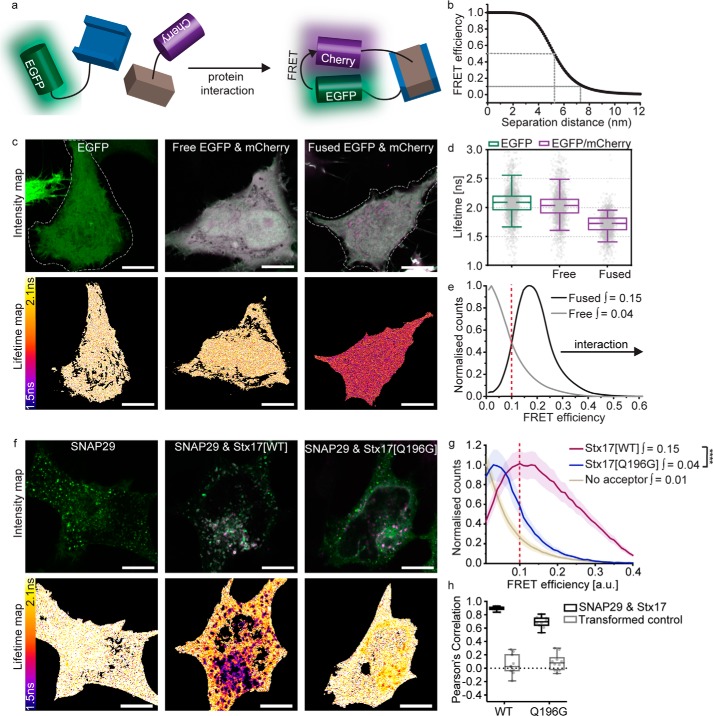
**FLIM-FRET confirms Stx17 and SNAP29 interact in autophagosomes via their SNARE motifs.** *a*, FLIM-FRET can be used to probe protein interactions *in situ*. During protein interaction, their fluorophore labels are held in close proximity, allowing FRET to occur between donor (EGFP) and acceptor (mCherry), resulting in a reduced lifetime of the donor and acceptor–stimulated emission. *b*, theoretical relationship between FRET efficiency and separation distance for EGFP and mCherry, demonstrating the sensitivity of FRET efficiencies above 0.1 to protein proximities < 8 nm. *c*, intensity and fluorescence lifetime images of unfused, soluble EGFP, co-expressed unfused EGFP and mCherry or directly fused EGFP-mCherry. FLIM analysis was restricted to individual cells; a manually drawn ROI (indicated with a *dotted line*) excluded neighboring cells where present. *d*, fluorescence lifetime box plots derived from single pixel analysis of the data presented in *c*. Box-and-whisker plots represent the median (*central line*), 25th and 75th quartile (*box*), and 5th and 95th quartile (*whiskers*). *e*, histogram of the normalized single-pixel FRET efficiency counts for free or fused EGFP-mCherry. Integrated values above 0.1 are given; protein proximity causes a right shift in FRET efficiency and an increase in the integral. *f*, intensity and lifetime maps of EGFP-SNAP29 alone or co-expressed with either Stx17[WT] or Stx17[Q196G] in rapamycin-treated HeLa cells. *g*, mean single-pixel FRET efficiency histograms derived from the data set presented in *f*, enveloped by their S.E. Integrated values above 0.1 were tested for statistical significance using a Mann–Whitney test (*n* = 4 cells). *h*, a box plot of Pearson's correlation coefficients to quantify the fluorescence colocalization between the mutant or WT Stx17 and SNAP29 (*n* = 4). *Scale bars*, 10 μm throughout. ****, *p* < 0.0001.

Fluorescence colocalization studies can be used to observe the presence of two proteins on the same cellular structures, but not their direct association. Protein interactions can instead be inferred using FLIM-FRET, enabling us to verify, *in situ*, the autophagosomal SNARE complex. As illustrated by the *cartoon* in Fig. 2*a*, FRET is the nonradiative transfer of energy from a donor fluorophore (EGFP) to an acceptor (mCherry), requiring sustained proximity of the fluorophore pair, typically driven by protein interaction. The occurrence of FRET causes the donor fluorescence to decay more rapidly; quantification of this property as 1/*e*, termed its fluorescence lifetime, provides a robust metric for proximity. As protein proximity increases, the FRET efficiency increases, causing fluorescence lifetime to decrease.

For the FRET pair EGFP and mCherry, 50% FRET efficiency theoretically occurs at 5.24-nm separation (33) and requires sub-8-nm proximities for FRET to occur, evidenced by FRET efficiencies of 0.1 and greater (Fig. 2*b*). EGFP has a similar median fluorescence lifetime of 2.09 and 2.04 ns when expressed alone or co-expressed with mCherry, respectively; however, this is reduced to 1.73 ns if the two fluorophores are directly fused by a 12-amino acid linker (Fig. 2, *c* and *d*). Forced proximity of mCherry therefore causes a shift away from zero of single-pixel FRET efficiencies (Fig. 2*e*).

It is important to note that fluorescence lifetime measurements present intrinsic variation due to their sensitivity toward the local microenvironment, the cellular interaction pattern, and the photon statistics used to inform the fit. As further detailed under “Experimental procedures,” we therefore instead compare single-pixel FRET efficiency histograms of each cell to detect even localized interactions, removing low-count pixels that produce unreliable fluorescence decay fits. We found that the integral of these curves between 0.1 and 1 provides a useful single metric to describe the occurrence of FRET. As changes in FRET efficiency are typically slight, it can be troublesome to determine their significance. We therefore present a statistical comparison of these integral values as a nonbiased means to conclude protein proximity.

FLIM-FRET analysis of EGFP-SNAP29 demonstrates a reduction in the EGFP fluorescence lifetime when co-expressed with mCherry-Stx17 (Fig. 2*f*), manifesting in an increased FRET efficiency integral similar to fused EGFP-mCherry (Fig. 2*g*). The change in fluorescence lifetime is restricted spatially to punctate structures (Fig. 2*f*), despite the presence of a soluble population of both proteins in the cytosol (7, 34) and the evident SNARE-binding promiscuity of SNAP29 (34). This indicates that an unknown inhibitory mechanism likely prevents ectopic association.

It is possible that the increased FRET is due to short mean distances between highly concentrated overexpressed proteins and not caused by specific protein interactions. To exclude this possibility, a mutant Stx17 predicted not to form SNARE interactions was prepared by mutation of the conserved zero-layer glutamine residue (mCherry-Stx17[Q196G]), known to be essential for syntaxin function (35, 36). Although this mutant interacted significantly with EGFP-SNAP29, it produced significantly lower FRET efficiencies than we saw with WT Stx17 (Fig. 2*g*), confirming a SNARE domain–mediated association of WT Stx17 and SNAP29. Mutant Stx17 does, however, still significantly colocalize with SNAP29 (Fig. 2*h*), which, together with the reduced but significant FRET detected, may indicate a weakened or conformationally altered association.

**Figure 3.**
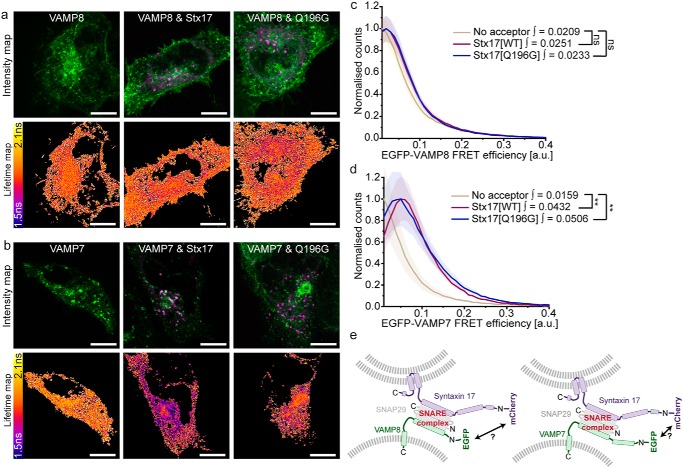
***In situ* FLIM-FRET identifies VAMP7 as a heterodimer-dependent interaction partner of Stx17.** *a* and *b*, intensity images and their corresponding fluorescence lifetime maps probing changes in lifetime and proximity between donor-only or donor and acceptor samples in rapamycin-treated cells. *Scale bars*, 10 μm. Shown is EGFP-VAMP8 (*a*) and EGFP-VAMP7 (*b*) expressed alone or co-expressed with either WT mCherry-Stx17 or mCherry-Stx17[Q196G]. *c* and *d*, mean single-pixel FRET efficiency histograms, enveloped by S.E., derived from the data sets presented in *a* and *b*, respectively. Statistical significance was tested using a Mann–Whitney test to compare integral values > 0.1 (*n* = 7–10 cells). *e*, *schematic* demonstrating the differing R-SNARE compositions, which have an unknown impact on complex structure and fluorophore separation distance. *ns*, nonsignificant (*p* ≥ 0.05); **, *p* = 0.0005–0.005.

FLIM-FRET analyses of EGFP-labeled endolysosomal R-SNAREs and mCherry-Stx17 report sub-8-nm proximity with EGFP-VAMP7, but not with EGFP-VAMP8 (Fig. 3, *a–d*). Surprisingly, expression of the Stx17[Q196G] mutant in place of WT Stx17 did not significantly reduce the FRET efficiencies observed between VAMP7 and Stx17. Instead, FRET efficiencies similar to the residual SNAP29 and Stx17[Q196G] interaction were recorded, which may point to the presence of a stabilizing regulatory protein.

As VAMP7 has an N-terminal longin domain that is not present on VAMP8, it is possible that VAMP8 also interacts with the autophagosomal SNARE complex *in situ* but outside the 8-nm range required for FRET detection (Fig. 3*e*). However, given that VAMP7 signal alone correlates with Stx17 and demonstrates a fusion-dependent correlation with LC3 (Fig. 1 and Fig. S3, respectively), we contend that VAMP7 is an alternative, if not the dominant, R-SNARE involved in autophagosome clearance.

### Autophagosomal SNARE interactions are modulated by Stx17 serine 2 phosphorylation

Given that SNARE proteins are highly reactive, a regulatory mechanism must exist to prevent ectopic heterodimer formation and uncontrolled fusion. This regulation could be either inhibitory or obligatory (*i.e.* where an additional factor is essential to permit SNARE complex formation). SNARE-mediated autophagosome clearance is reportedly promoted at the fusion site by SNARE bundle–stabilizing proteins, including HOPS-associated VPS33A (16) and Atg14 (37). We hypothesized that an as yet unknown inhibitory mechanism may also exist to prevent premature SNARE associations with Stx17.

**Figure 4.**
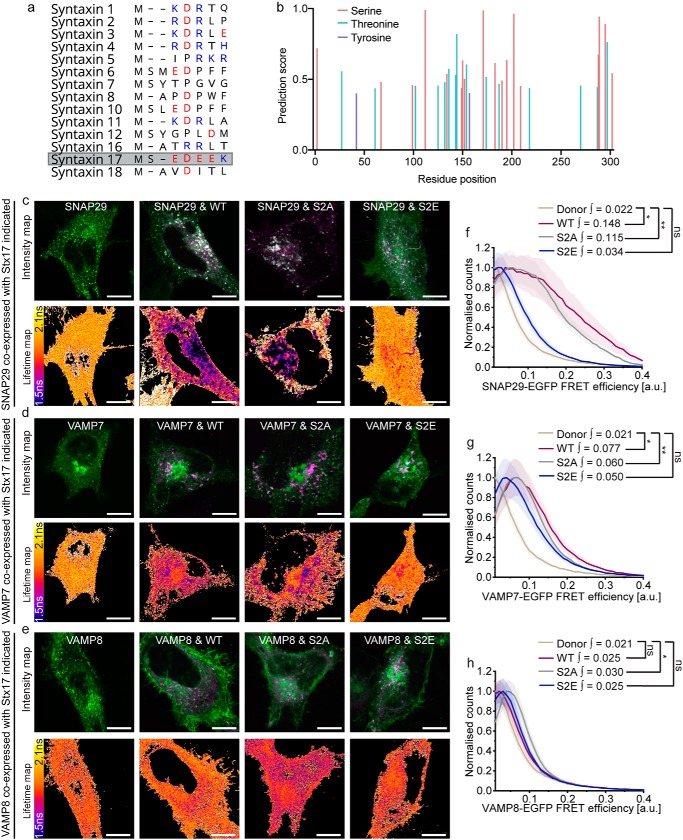
**Stx17 N-terminal phosphoserine modulates SNARE complex formation.** *a*, an alignment of the N-peptide sequences of human syntaxin family proteins; negatively and positively charged residues are indicated in *red* and *blue*, respectively. Stx17 (*gray*), encodes a highly negative N terminus. *b*, NetPhos 3.1 phosphorylation site prediction scores for each residue of Stx17 indicates consensus for serine 2 phosphorylation. *c–e*, intensity and fluorescence lifetime maps of rapamycin-treated HeLa cells expressing EGFP-SNAP29 (*c*), EGFP-VAMP7 (*d*), or EGFP-VAMP8 (*e*) alone or alongside mCherry-fused Stx17[WT], Stx17[S2A], or Stx17[S2E] as indicated. *Scale bars*, 10 μm. *f–h*, mean single-pixel FRET efficiency histograms, enveloped by their S.E., derived from the data sets presented in *c–e* respectively; integral values describe efficiencies between 0.1 and 1. Statistical significance was tested using a Mann–Whitney test to compare single-cell integral values > 0.1 (*n* = 9–14 cells). *ns*, nonsignificant (*p* ≥ 0.05); *, *p* = 0.005–0.05; **, *p* = 0.0005–0.005.

Syntaxin N-peptides can have a regulatory interaction with domain 1 of their cognate SM protein. However, the noncanonical sequence of this region in VPS33A led to the notion that it does not engage in this manner (20, 38). Similarly, Stx17 is unusual among syntaxin family members for its highly charged N-peptide; it consists of a string of four negative residues flanked by a predicted phosphoserine site with consensus for CK2 (39) (Fig. 4, *a* and *b*). Stx17 serine 2 phosphorylation, which has been confirmed in phosphoproteome studies (40), would have the effect of further increasing the net negative charge in this molecular region.

We speculated that the negative N-peptide patch of Stx17 may enable a novel means of regulation that is modulated by serine 2 phosphorylation, similar to serine 14 phosphoregulation of syntaxin 1a (41). Indeed, truncation of the N-terminal peptide has recently been shown to block autophagic flux (42). We therefore developed phoshonull and phosphomimetic mutants of Stx17, Stx17[S2A] and Stx17[S2E], respectively, to ascertain their impact on formation of the SNARE complex *in situ* using our FLIM-FRET assay. No alteration in the expression pattern of any of the mutants was observed, meaning that their availability to binding partners was also unaffected. When FLIM-FRET was performed with these mutants to determine their interaction with SNAP29, we observed a profound, spatially restricted autophagosomal reduction in FRET only for the phosphomimetic mutant (S2E; Fig. 4, *c* and *d*).

A similar effect was observed for VAMP7, with the Stx17[S2E] mutant showing no significant increase in FRET efficiency when compared with donor-only values (Fig. 4, *e* and *f*). By comparison, WT Stx17 and Stx17[S2A] both presented significant FRET efficiencies in the presence of either SNAP29 or VAMP7, indicating that increased negative charge at Stx17 serine 2 decreases SNARE complex formation, providing a putative inhibitory mechanism of autophagy regulation. Notably, the same assay carried out with VAMP8 demonstrated significantly higher FRET efficiencies only for the phosphonull mutant, Stx17[S2A], suggesting that loss of this phosphoregulatory mechanism leads to uncontrolled SNARE complex formation (Fig. 4, *g* and *h*).

### Multimodal regulation of Stx17 by VPS33A provides an autophagy master-switch

It is hard to conceive how a single charge mutation in the N-peptide of Stx17 alone could completely abolish detectable interaction with SNAP29 without the intervention of a modulating third party. Indeed, its N-peptide location hints at a second mode interaction with an SM protein, akin to syntaxin–SM proteins elsewhere in the cell (10, 43, 44). Importantly, however, an inhibitory role for VPS33A has been ruled out based on the structural absence of an N-peptide–binding pocket (38) that appears to preclude interaction with monomeric syntaxin *in vitro* (20).

It is known that the SM protein VPS33A promotes autophagosome clearance, and its knockdown therefore blocks autophagic flux (16, 19). Although the mechanism of action has never been established, the presence of a four-α-helical binding groove in VPS33A (45) hints that it can accommodate the SNARE bundle to physically stabilize cognate SNARE associations, as seen for other SNARE–SM pairings (10).

**Figure 5.**
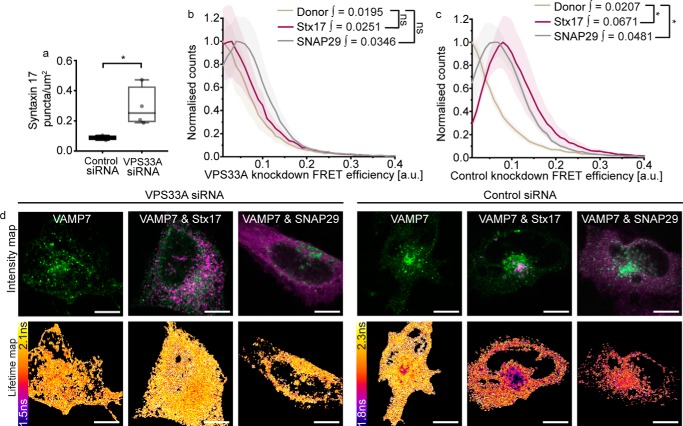
**VPS33A promotes fusion by stabilizing the SNARE bundle.** *a*, box plot of Stx17-positive puncta concentration per μm^2^ in rapamycin-treated HeLa cells, demonstrating accumulation of autophagosomes upon VPS33A knockdown (VPS33A siRNA) when compared with a nontargeting negative control knockdown (control siRNA). Significance was tested using an unpaired two-sample *t* test (*n* = 4). *b–d*, FLIM-FRET analysis of rapamycin-treated HeLa cells expressing EGFP-VAMP7 alone or alongside either mCherry-Stx17 or mCherry-SNAP29. Mean single-pixel FRET efficiency histograms for VPS33A siRNA–treated samples (*b*) and negative control siRNA-treated samples (*c*), both enveloped by their S.E. FRET efficiency integral values >0.1 were tested for statistical significance using a Mann–Whitney test (*n* = 4 cells). *d*, representative intensity and fluorescence lifetime maps. *Scale bars*, 10 μm. *ns*, nonsignificant (*p* ≥ 0.05); *, *p* = 0.005–0.05.

To verify this, we recapitulated VPS33A knockdown studies to determine whether its loss causes destabilization of the autophagosomal SNARE bundle. A FLIM-FRET assay of ternary complex formation was used to assess the proximity of both Stx17 and SNAP29 with VAMP7 in control and VPS33A knockdown cells. Consistent with published work (16), significant Stx17 puncta accumulation was observed in VPS33A siRNA-treated cells (Fig. 5*a*). This accumulation coincided with loss of the EGFP-VAMP7 FRET detected in control knockdown cells when mCherry-Stx17 or mCherry-SNAP29 is present (Fig. 5, *b–d*), validating for the first time *in situ* that VPS33A is required to promote autophagosome clearance by facilitating SNARE bundle formation (16).

**Figure 6.**
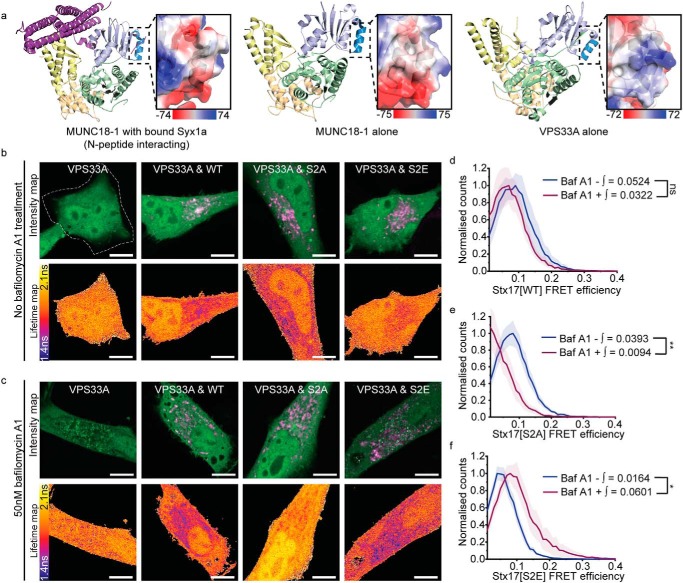
**A phosphorylation master-switch in Stx17 regulates its interaction with VPS33A.** *a*, Cartoon diagrams of the structure of syntaxin 1a–bound Munc18–1 (*left*), Munc18–1 alone (*center*), and VPS33A alone (*right*). SM protein structure is *colored* by domain (domain 1 is *light blue* with *dark blue* highlighting the N-peptide–binding region; domain 2 is *green*; and domains 3a and 3b are *yellow* and *wheat*, respectively). *Magnified panels* show the electrostatic surface charge in kT/*e*_c_ of the indicated syntaxin N-peptide–binding region. *b* and *c*, intensity and fluorescence lifetime maps of rapamycin-treated HeLa cells expressing EGFP-VPS33A or co-expressed with mCherry-Stx17 in the absence (*b*) and presence (*c*) of bafilomycin A1. FLIM analysis was restricted to individual cells, and a manually drawn ROI (indicated with *dotted line*) excluded neighboring cells where present. *Scale bars*, 10 μm. *d–f*, corresponding mean single-pixel FRET efficiency histograms, enveloped by their S.E. and derived from EGFP-VPS33A fluorescence lifetimes in Stx17-puncta regions only. These panels compare changes to FRET efficiencies in the presence or absence of 50 nm bafilomycin A1 treatment with Stx17[WT] (*d*), Stx17[S2A] (*e*), or Stx17[S2E] (*f*) acceptors. FRET efficiency integral values > 0.1 were tested for statistical significance using a Mann–Whitney test (*n* = 6–10 cells). *ns*, nonsignificant (*p* ≥ 0.05); *, *p* = 0.005–0.05; **, *p* = 0.0005–0.005.

To address our hypothesis that VPS33A may additionally bind monomeric Stx17 in an inhibitory interaction, we first carried out a structural analysis to assess binding potential. As VPS33A lacks the partially conserved N-peptide–binding pocket that has been shown to mediate monomeric syntaxin interactions in other SM proteins (38), we speculated that it may instead bind the Stx17 N-peptide through charge interactions. We observed strikingly positive electrostatic surface potentials of VPS33A position analogous to the N-peptide–binding region in other family members (38) (Fig. 6*a*), which we suspect interacts with the negatively charged Stx17 N-peptide in a manner mediated by serine 2 phosphorylation. Indeed, structural analyses of the HOPS complex have confirmed that both the syntaxin N-peptide-binding region and the four-α-helical binding groove of VPS33A are exposed when HOPS-associated (45–47), which would therefore allow for multimodal regulation.

To define this interaction *in situ*, we temporally dissected the pathway by chemical inhibition of the fusion event using bafilomycin A_1_, an inhibitor of autophagy completion. This drug is known to inhibit lysosome acidification by antagonizing the vacuolar type H^+^-ATPase (V-ATPase) and has been shown to additionally inhibit autophagosome fusion (48), although whether and how this occurs remains under debate. The latter function is essential to dissect the fusion pathway, and by employing a long treatment protocol, previously shown to inhibit fusion (49), we successfully abolished the colocalization of VAMP7-positive endolysosomes with LC3-positive autophagosomes (Fig. S3). In our case, treatment thus provides a snapshot of pre-fusion interactions, dissecting these from the mix of pre- and post-fusion interactions observed when autophagic flux is active. FLIM-FRET confirmed an interaction between VPS33A and WT Stx17 *in situ* (Fig. 6, *b–d*). The change in fluorescence lifetime was localized to Stx17-positive puncta (Fig. 6, *b* and *c*), and FRET efficiencies in these regions were not significantly altered by the addition of baf A_1_ (Fig. 6*d*), indicative of an Stx17/VPS33A interaction that persists throughout autophagosomal fusion.

We repeated this assay with the phosphonull and phosphomimetic Stx17 mutants to differentiate our postulated inhibitory mode interaction of VPS33A/Stx17 from the SNARE bundle-stabilizing interaction of VPS33A. Importantly, these mutants produced opposite interaction phases with VPS33A, both changing significantly upon treatment with baf A_1_. Stx17[S2E], which cannot form a SNARE complex (Fig. 4) and therefore represents an inhibited form of Stx17, associated with VPS33A significantly more in baf A_1_–treated cells, representing pre-fusion association (Fig. 6*f*). Conversely, Stx17[S2A] associated with VPS33A significantly more in fusion-competent cells (Fig. 6*e*), suggesting that dephosphorylation of serine 2 relieves Stx17 of an inhibitory mode interaction with VPS33A when fusion is needed. Notably, these differences in mutant Stx17 FRET are specific to VPS33A; Atg14, another promoter of autophagosomal SNARE assembly (37), appears to interact with all Stx17 mutants (Fig. S4).

**Figure 7.**
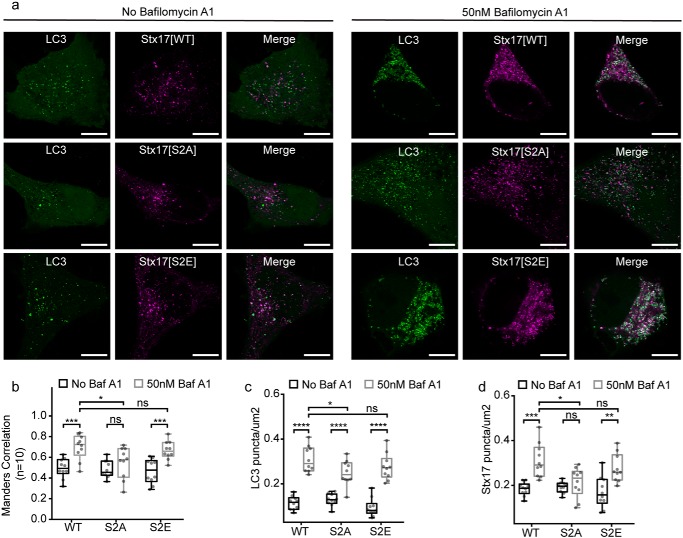
**Stx17 N-peptide phosphorylation inhibits ectopic autophagosome clearance.** *a*, representative single-channel and merged images of autophagic HeLa cells expressing EGFP-LC3 and the variant of mCherry-Stx17 are indicated. Both fusion-competent cells (*left panels*) and fusion-incompetent baf A_1_–treated cells (*right panels*) are displayed. *Scale bars*, 10 μm. *b* and *c*, box plots of EGFP-LC3 (*b*) or mCherry-Stx17 (*c*) puncta concentration per cell in the presence or absence of baf A_1_. *d*, box plots of Manders correlation coefficient to quantify colocalization between channels in *a*. Statistical significance tested with unpaired two-sample *t* tests (*n* = 10). *ns*, nonsignificant (*p* ≥ 0.05); *, *p* = 0.005–0.05; **, *p* = 0.0005–0.005; ***, *p* = 0.0001–0.0005; ****, *p* < 0.0001.

To determine the functional implications of these interactions, we assessed how expression of each mutant affects autophagic flux. As EGFP (but not mCherry) is hydrolyzed following autophagosome clearance (29), EGFP-LC3 puncta number provides a useful indicator of flux. Chemically blocking clearance with baf A_1_ led to LC3 puncta accumulation in cells expressing each mutant, confirming that all cells were fusion-competent (Fig. 7, *a* and *b*), perhaps assisted by background endogenous Stx17.

However, a modest but significant reduction in the scale of this accumulation was noted in cells expressing Stx17[S2A] when compared with WT Stx17 (Fig. 7*b*). Importantly, there was no significant increase in the LC3 puncta number of Stx17[S2A]-expressing cells prior to baf A1 treatment when compared with Stx17[WT]-expressing cells, which would have indicated blocked fusion. Reduced LC3 puncta accumulation upon baf A_1_ treatment is therefore not a symptom of reduced fusogenicity but reduced autophagosome availability or circumvention of the drug-induced fusion block. Such an effect could be caused by uncontrolled and/or ectopic autophagosome clearance. In support of this, Stx17[S2A] puncta do not accumulate when fusion is blocked (Fig. 7*d*), suggesting clearance independent of regulated autophagosome–endolysosome fusion.

An alternative interpretation may be that Stx17[S2A] recruitment to the autophagosome is specifically disrupted in the baf A_1_ state. In this case, we would expect no change to the LC3 puncta number when compared with WT Stx17 and a reduction in LC3/Stx17[S2A] puncta colocalization upon baf A_1_ treatment, neither of which are observed (Fig. 7, *b* and *c*).

Taken together with our interaction data (Figs. 4–6), we contend that loss of the regulatory step in Stx17[S2A] permits early and ectopic clearance of autophagosomes. We speculate that by disengaging SNARE complex formation from SM protein action, negative regulation is alleviated, enabling uncontrolled, premature, and ectopic fusion to proceed with any compatible SNARE pairings in the endomembrane system. We therefore identify Stx17 serine 2 as an essential spatial and temporal decision point for autophagosomal fusion to proceed.

## Discussion

In this paper, we sought to address both the lack of *in situ* data defining the composition of the autophagosomal SNARE complex and the gaps in our knowledge of how autophagosome clearance is regulated. Using high-resolution FLIM-FRET to detect SNARE interactions within HeLa cells and a series of mutant Stx17 analyses, we now propose a revised model of autophagosome clearance.

**Figure 8.**
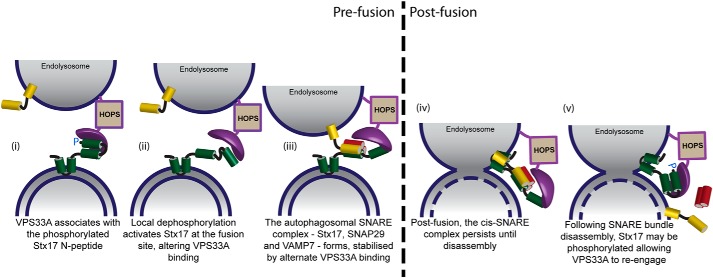
**Proposed model of Stx17 interaction dynamics with VPS33A.** Syntaxin 17 (*green*) and VPS33A (*purple*) interact via two distinct binding modes switched by the phosphorylation status of Stx17 serine 2. We propose that prior to fusion, VPS33A domain 1 associates with the phosphorylated Stx17 N-peptide (*i*); the Stx17 N-peptide is locally dephosphorylated at the fusion site, which alters VPS33A binding and enables SNARE bundle formation, providing a final stage regulatory mechanism (*ii*); and SNAP29 and VAMP7 may now associate with Stx17, forming a SNARE bundle that is stabilized by an alternate VPS33A association and that drives membrane fusion (*iii*). Following fusion, the *cis*-SNARE bundle persists until actively disassembled (*iv*), following which, we suspect that Stx17 serine 2 is phosphorylated once more, allowing re-engagement of the VPS33A domain 1 with the Stx17 N-peptide to deter further fusion events (*v*).

Our model (overviewed in Fig. 8) contends that Stx17 has both a SNARE-binding and inhibited form, dependent on its N-terminal phosphorylation status (Fig. 4), each of which associates differently with VPS33A (Figs. 5 and 6). The nonreactive, “phosphorylated” form of Stx17 predominantly interacts with VPS33A prior to fusion (Fig. 6), seemingly preventing ectopic fusion (Fig. 7). When fusion is allowed to proceed, we instead observe preferential engagement of VPS33A with “dephosphorylated” Stx17 (Fig. 6), likely via alternate binding and stabilization of the SNARE bundle (Fig. 5) within its four-helical binding groove. The composition of this SNARE bundle appears to be Stx17, SNAP29, and VAMP7 (Figs. 1–3). For this change in fusogenicity to occur, Stx17 serine 2 must be locally dephosphorylated by an as yet undefined phosphatase, thereby releasing the N-peptide interaction with VPS33A.

This mechanism provides a previously unknown but essential final checkpoint for fusion. However, it remains to be determined whether VPS33A actively inhibits phosphorylated Stx17, stabilizes a self-inhibitory conformation of Stx17, or is required to recruit and transition Stx17 from an inactive to a reactive form.

Notably, and contrary to our finding that VAMP7 is the dominant R-SNARE, knockdown studies conducted by other researchers found that VAMP8, but not VAMP7, knockdown affected xenophagic flux (50). It is unclear whether this is due to residual SNARE expression, known to be sufficient for continued fusion (51), or if VAMP8 may also contribute to the autophagic SNARE bundle, perhaps undetected here due to a different conformation extending beyond the 8-nm detection limit of this assay. It therefore remains to be determined whether VAMP7 is the sole autophagy R-SNARE or if it performs a complementary function to VAMP8. Indeed, VAMP7 has previously been implicated in discrete steps, including autophagosome biogenesis (52) and autophagosome–endosome fusion, to form the intermediate amphisome (21).

A central challenge in SNARE biology is to understand the interplay of the varied binding interactions of SM protein regulators. SM proteins can promote or inhibit SNARE-mediated fusion dependent on the binding mode (43, 44), but the observed associations and their outcomes appear to vary for different syntaxin–SM pairings (38). To reconcile these differences, it was recently proposed that two classes of SM proteins exist: class I, which have primary affinity for monomeric syntaxin and are characterized by N-peptide binding, and class II, which primarily bind the SNARE complex and lack N-peptide–binding capability (20). Our findings that VPS33A, the exemplar class II SM protein, has a structurally divergent but functionally analogous N-peptide–binding patch, necessitate revision of this model.

Based on the data presented and their similarities with other mammalian syntaxin–SM interaction dynamics (53, 54), we put forward a unified model of SM regulation where, *in situ*, N-peptide interactions are conserved across syntaxin–SM pairings, fulfilling a recruitment and tethering function. In the case of syntaxin 1a, N-peptide tethering is sustained to aid binding mode transitions (55), which, given the changes in FRET efficiency between phosphonull and phosphomimetic N-peptide mutants (Fig. 6), is likely accomplished by other means for Stx17. Indeed, VPS33A-associated HOPS components, VPS11 and VPS18, have been shown to associate with the Stx17 Habc domain (20).

Our findings have additional implications for the regulation of autophagosomal clearance. First, VAMP7 notably differs from VAMP8 in having a regulatory longin domain, consistent with the autophagosomal R-SNAREs identified in *D. melanogaster* and *S. cerevisiae* (23, 56). This structural conservation may suggest an additional analogous regulatory mechanism that is as yet unexplored. Second, the evident inhibitory role of phosphorylation for autophagosome clearance may couple this process to intracellular energy levels based on ATP availability. If this is indeed the case, it would provide a fast feedback mechanism to prevent the unnecessary degradation of cellular material upon a return to nutrient-replete conditions, bypassing the delayed onset of TORC1 reactivation (57).

## Experimental procedures

### Cell culture, transfection, and fixation

The HeLa cells used throughout this study were maintained at 37 °C and 5% CO_2_ in Dulbecco's modified Eagle's medium supplemented with 100 units/ml penicillin, 100 μg/ml streptomycin, 10% heat-inactivated fetal bovine serum, 1× Glutamax, and 1 mm sodium pyruvate. Cells were cultured on poly-d-lysine hydrobromide-coated glass coverslips and transfected using Turbofect Transfection Reagent (Thermo Fisher Scientific) prior to fixation with 4% paraformaldehyde and 0.1% glutaraldehyde. Samples were treated with 50 mm ammonium chloride to reduce free aldehyde autofluorescence and mounted with Mowiol 4-88. In the case of knockdown experiments, siRNA transfection was carried out using Lipofectamine RNAiMAX (Thermo Fisher Scientific) 48 h prior to DNA transfection.

### Plasmids

All SNARE proteins used in this study were N-terminally tagged. pEGFP- and pmCherry-C1-Stx17, pEGFP- and pmCherry-C1-SNAP29, pEGFP-C1-VPS33A, and pEGFP-C1-VAMP8 were generated by ligating the PCR-isolated gene of interest from plasmids kindly provided by other laboratories (7, 16) (Addgene, 45909, 45923, 67022 and 45919) into the Clontech vector backbone. The mutant variants of pmCherry-C1-Stx17 (S2A, S2E, and Q196G) were obtained by site-directed mutagenesis using the QuikChange II site-directed mutagenesis kit (Agilent). pEGFP-C2-LC3 (58) (Addgene, 24920), pEGFP-C2-Atg14 (59) (Addgene, 21635), and pEGFP-C1-VAMP7 (60) (Addgene, 42316) were obtained from other groups, and the former was used to generate pmCherry-C2-LC3 and pEGFP-mCherry-C2-LC3 by restriction and ligation. pEGFP-N3 and pmCherry-N1 (Clontech, discontinued) were used for FLIM-FRET control work along with pCDNA3.1-EGFP-mCherry, a fusion of two fluorescent proteins separated by a 12-amino-acid linker, which was generated by isolation of the fusion protein from previously described pGEX-KG_EGFP-mCherry (55) and ligation into the pCDNA3.1 vector backbone.

### siRNA

VPS33A knockdown was achieved using VPS33A stealth siRNA (Thermo Fisher Scientific, HSS127975) with the following antisense sequence: 5′-AUGAGAUCUAAGCUGUACUCCUCCC-3′. Control knockdowns used siGENOME Non-Targeting siRNA Pool 2, which is designed to target no known human genes (Dharamacon, D-001210-02).

### Autophagy assays

Autophagy was induced in cells by treatment with 160 nm rapamycin (Thermo Fisher Scientific, PHZ1235) in normal growth medium for 1 h at 37 °C and 5% CO_2_ prior to fixation. To assay for autophagic activity based on puncta accumulation upon inhibition of autophagosome–endolysosome fusion, cells were additionally treated where described with 50 nm bafilomycin A_1_ (Sigma-Aldrich, B1793) for 24 h under normal culture conditions. Puncta accumulation was quantified per cell using the Spot Detector plugin (61) within the open source software, ICY version 1.8.4.0 (62).

### Colocalization image acquisition and quantification

Dual-channel intensity images were acquired using a Leica SP5 SMD confocal laser-scanning microscope (CLSM) equipped with a ×63/1.4 numeric aperture HCX PL Apo oil immersion objective lens. Samples were excited sequentially with 488- and 561-nm CW lasers, and emission was detected via internal photomultiplier tubes (Hamamatsu, R9624); channel alignment was experimentally validated (Fig. S2). Images were Nyquist-sampled with a 40-nm pixel size and processed by image deconvolution using a theoretical point spread function (Huygens Software, SVI). Additionally, a rolling-ball background subtraction (Fiji) and thresholding to exclude nonpunctate regions were carried out prior to Manders correlations. The Coloc 2 plugin (Fiji) (63) was used for all colocalizations. Negative control colocalization analyses were carried out by running the same algorithm on a fluorophore-dense 11 × 11-μm region of interest with a 90° transformation of the mCherry channel only. Box-and-whisker plots presented throughout show the minimum and maximum values (*whiskers*), 25th and 75th quartiles (*box*), and the median value (*line*); all data points are also indicated (*dots*).

### Stimulated emission depletion microscopy (STED)

Continuous-wave (CW) gated STED microscopy was performed with a ×100/1.4 numeric aperture HCX PL Apo oil immersion objective lens on a Leica SP5 SMD CLSM with STED capability. EGFP was excited using a white light laser tuned to 488 nm with an 80-MHz pulse rate. Refinement of the point spread function was accomplished by concurrent depletion with an aligned CW 592-nm depletion laser. Emission wavelengths were isolated as for standard CLSM and detected with a time-gated Leica HyD hybrid detector synchronized to the excitation pulse. As the efficiency of depletion increases with depletion time (64), time-gated detection between 0.5 and 12 ns was employed to ensure optimal depletion prior to detection. Images were acquired with a 0.015-μm pixel size to ensure Nyquist sampling.

### FLIM data acquisition

Fluorescence lifetime images were acquired on a Leica SP5 SMD confocal laser-scanning microscope fitted with a time-correlated single photon counting module (PicoHarp 300) using a ×63/1.4 numeric aperture HCX PL Apo oil immersion objective lens. The donor EGFP was excited using a tunable white light supercontinuum laser operating at 488 nm and pulsing at 40 MHz. Emission was detected with an external single-photon avalanche diode (MicroPhoton Devices). Recordings were integrated to achieve a maximum pixel count of 10,000 photons, sufficient for accurate single-pixel fitting. Photon arrival times were recorded across 1600 time bins within the 25-ns window for each 161-nm pixel. Donor-only samples were prepared under identical conditions and alongside test samples. The donor controls were imaged on the same day as test samples to minimize as far as possible variation in fluorescence lifetimes caused by differences in fluorophore microenvironment.

### FLIM-FRET data analysis

Single-pixel fluorescence lifetime analyses were carried out with SymPhoTime version 5.4.4 (PicoQuant). Cells were analyzed individually and were defined using a manually drawn ROI if needed to exclude neighboring cells overlapping into the field of view (indicated with a *dotted line* in the figures). A model fluorescence decay curve was generated from all pixels across a cell of interest and fitted bi-exponentially using the maximum-likelihood estimation method. Bi-exponential fitting was required to accurately fit EGFP decays both in the absence and the presence of an acceptor (Fig. S5). The resulting fit parameters were subsequently used to guide single-pixel fitting. The output values for amplitude, lifetime, and intensity were exported for graphical analysis, and the generation of amplitude-weighted average fluorescence lifetime maps, referred to in the text as “lifetime maps,” was determined as follows,

(Eq. 1) $$\tau_{\text{amp}}=\frac{A_{1}\tau_{1}+A_{2}\tau_{2}}{A_{1}+A_{2}}$$

where *A* and τ are the amplitude and lifetime of the indicated exponent. To improve the reliability of the fit statistics, pixels with < 1000 photon counts were excluded, as were fits reporting negative amplitudes or lifetimes. FRET efficiency was calculated as the following,

(Eq. 2) $$\text{FRET efficiency}=1-\left(\frac{\tau_{DA}}{\tau_{D}}\right)$$

per pixel of the donor-acceptor image (τ*_DA_*) using the median amplitude-weighted fluorescence lifetime of all analyzed donor-only pixels (τ*_D_*) per data set. Single-pixel FRET efficiency histograms were generated per cell, incorporating thousands of single-pixel values. This is presented throughout as the mean number of counts per bin across all analyzed cells (cell number specified as *n* in legends), enveloped by the S.E. Statistical significance was determined by comparing the 0.1–1 integral values, indicative of the occurrence of FRET, of the individual curves of each acquired field.

### Statistics

Statistical analyses were carried out using either GraphPad Prism version 7 or OriginPro. Statistical significance is presented throughout as follows: *ns*, nonsignificant (*p* ≥ 0.05); *, *p* = 0.005–0.05; **, *p* = 0.0005–0.005; ***, *p* = 0.0001–0.0005; ****, *p* < 0.0001.

## Author contributions

R. S. S., D. M. K., P. A. D., and R. R. D. investigation; R. S. S. and R. R. D. writing-original draft; R. S. S. and A. R. D. visualization; P. A. D. methodology; R. R. D. resources; R. R. D. and R. S. S. writing-review and editing.

## Supplementary Material

Supporting Information

## Footnotes

4 The abbreviations used are:

SNARE: soluble NSF attachment protein receptor
FLIM: fluorescence lifetime imaging microscopy
Stx17: syntaxin 17
SM: Sec1/Munc18
STED: stimulated emission–depletion
gSTED: gated STED
baf A_1_: bafilomycin A1
HOPS: homotypic fusion and vacuole protein sorting
EGFP: enhanced GFP
CLSM: confocal laser-scanning microscope
ROI: region of interest.
