# Supplementary material for: A VPS33A-binding motif on syntaxin 17 controls autophagy completion in mammalian cells
Source: J Biol Chem. 2019 Jan 17;294(11):4188–201. doi: 10.1074/jbc.RA118.005947 (PMC6422071; doi:10.1074/jbc.RA118.005947)
Supplement: Supporting Information [file supp_294_11_4188__index.html]

A VPS33A-binding motif on syntaxin 17 controls autophagy completion in mammalian cells — A novel syntaxin 17 regulatory motif controls autophagy — Supporting Information 

# A VPS33A-binding motif on syntaxin 17 controls autophagy completion in mammalian cells

## Supporting Information

- Supporting Information (to be published online) - Supplementary figures
