# Supplementary material for: A VPS33A-binding motif on syntaxin 17 controls autophagy completion in mammalian cells
Source: J Biol Chem. 2019 Jan 17;294(11):4188–201. doi: 10.1074/jbc.RA118.005947 (PMC6422071; doi:10.1074/jbc.RA118.005947)
Supplement: Supporting Information [file supp_RA118.005947_140854_1_supp_269607_plg294.pdf]

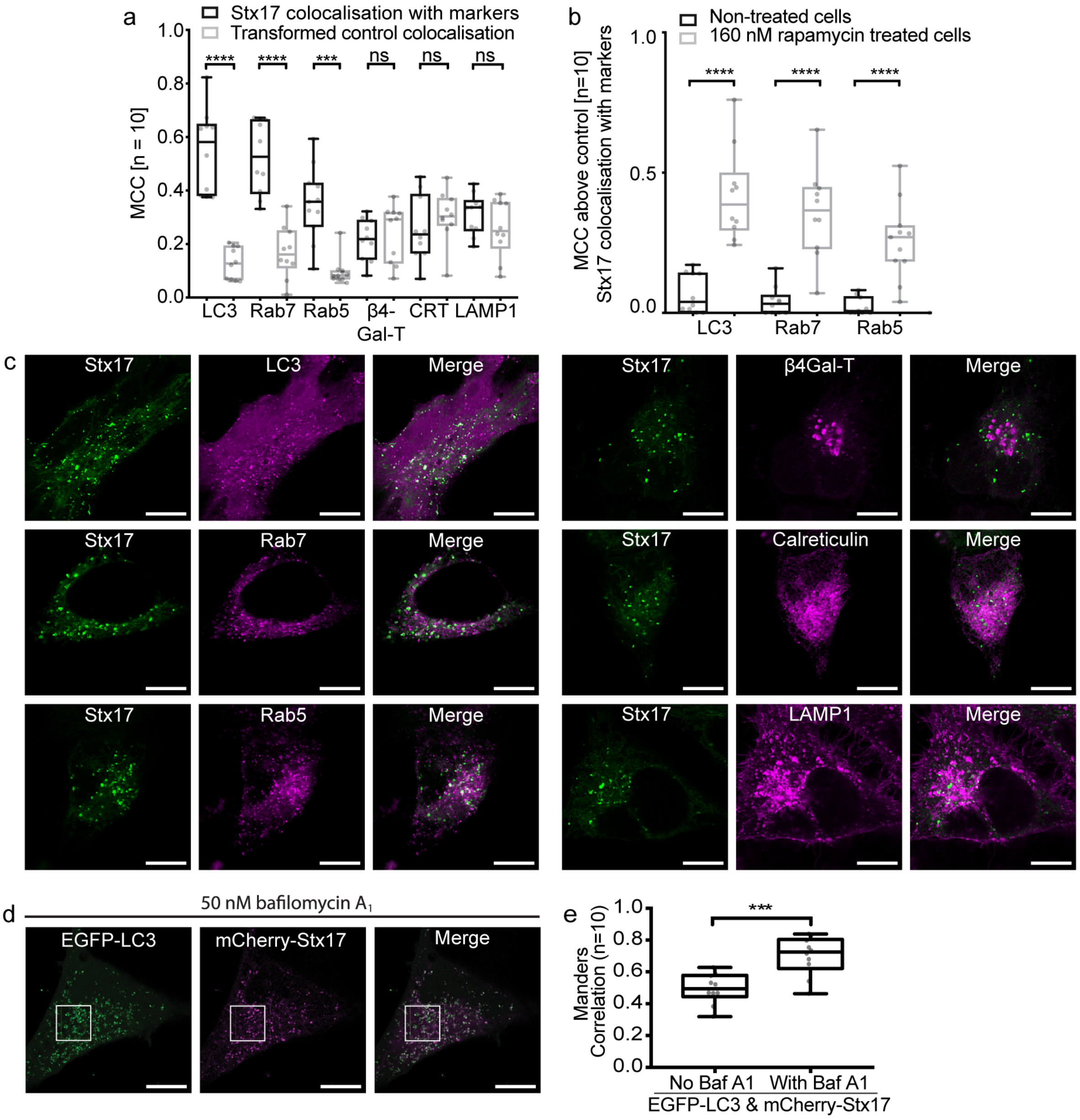

Supplementary figure 1 Stx17-EGFP resides on functional autophagosomes

(**a**) Manders correlation coefficient (MCC) of EGFP-Stx17 puncta colocal with the indicated mCherry-conjugated organelle markers when co-expressed in autophagic HeLa cells. LC3, Rab7, Rab5, β1,4-galactosyltransferase (β4Gal-T), calreticulin and LAMP1 identify autophagic compartments, late endosomes, early endosomes, Golgi structures, ER and lysosomes respectively. Statistical significance was tested with a paired two sample t-test, [n=10] (**b**) A barchart of the MCC values reported above the corresponding negative control value to compare colocalisation of Stx17 structures with autophagic and endosomal compartments in non-treated and rapamycin treated HeLa cells. Statistical significance was tested with an unpaired two-sample t-test, [n=10] (**c**) Representative single channel and merged images of autophagic HeLa cells co-expressing Stx17 and each of the organelle markers. (**d**) Single channel and merged images of autophagic HeLa cells co-expressing EGFP-LC3 and mCherry-Stx17 in the presence of bafilomycin A_1. (**c**) Box-and-whisker plots of Manders correlation coefficients to quantify the change in EGFP-LC3 and mCherry-Stx17 signal correlation upon bafilomycin A_1 treatment. Statistical significance tested using an unpaired student t-test, showing a significant increase in correlation upon inhibition of fusion with bafilomycin A_1. Scale bars are 10 µm throughout.

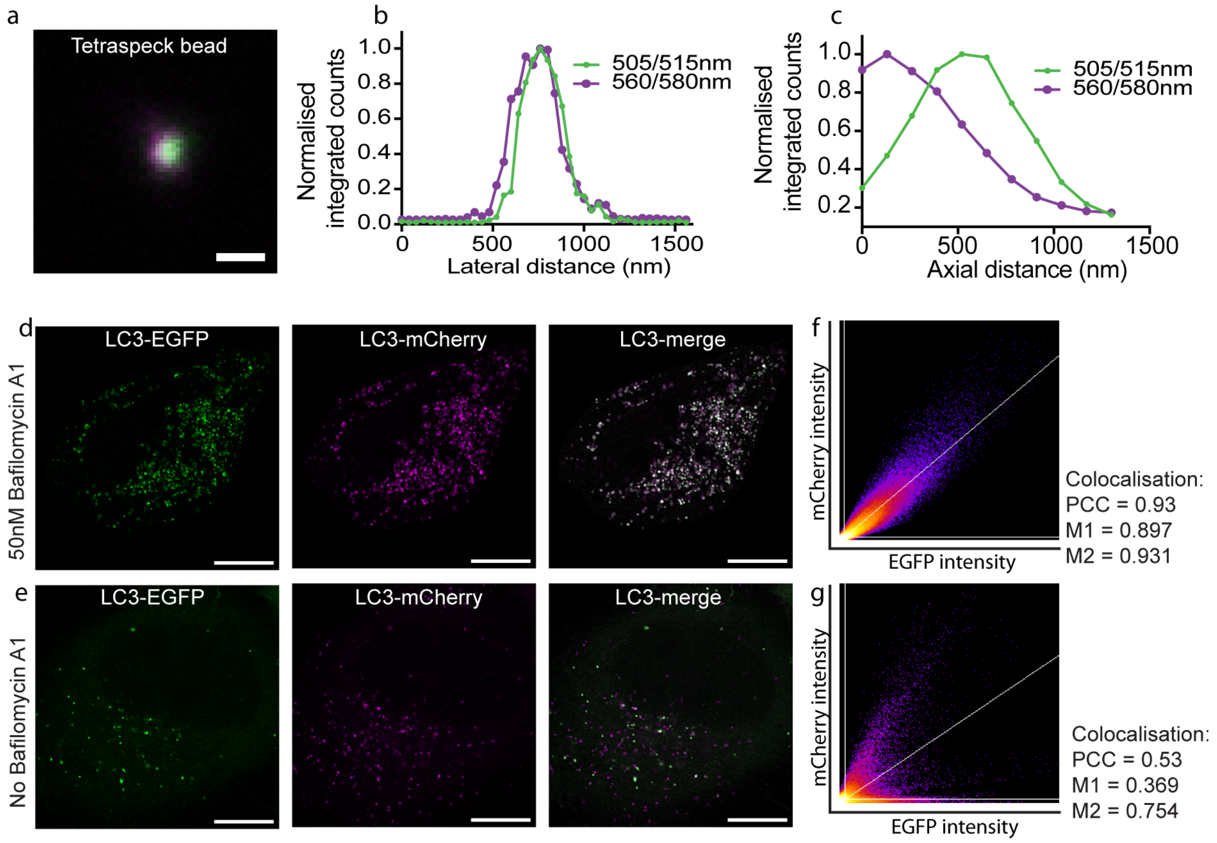

Supplementary figure 2 Fluorescence colocalisation acquisition and analysis

(**a**) A merged image of a single TetraSpeck bead with peak excitation and emission at 505 nm and 515 nm (green) or 560 nm and 580 nm (purple), scale bar 500 nm. (**b**) Lateral and (**c**) axial intensity profiles of the bead in (a). These indicate good lateral colocalisation but strong axial chromatic aberration, restricting colocalisation analyses to 2D datasets. (**d**) Intensity images of autophagic HeLa cells expressing dual-labelled LC3-EGFP-mCherry in the presence of bafilomycin A_1 to prevent lysosomal degradation of EGFP and (**e**) in the absence of bafilomycin A_1, scale bars are 10 µm. (**f**) A frequency scatter plot of the pixel intensities in (d), showing a linear relationship and (**g**) the same for (e), showing a non-linear relationship. As indicated, PCC reports high colocalisation for (d) but is more difficult to interpret for (e) where MCC demonstrates that as expected, only a subset of mCherry vesicles colocalise with EGFP (M1) while almost all EGFP vesicles colocalise with mCherry (M2).

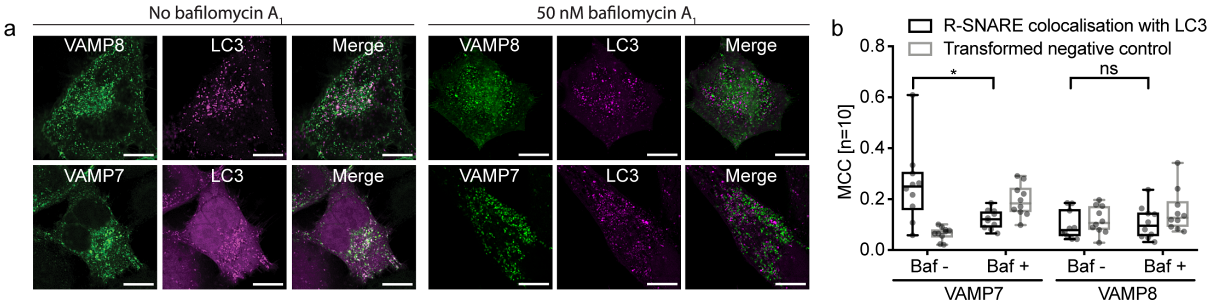

Supplementary figure 3 Fusion-dependent colocalisation of VAMP7 and LC3

(**a**) Representative single channel and merged images of autophagic HeLa cells co-expressing VAMP8 (top row) or VAMP7 (bottom row) with LC3, both in the presence (right) or absence (left) of the autophagosome-endolysosome fusion inhibitor, bafilomycin A_1. Scale bars are 10 µm. (**b**) A barchart comparing the fold increase in Manders correlation coefficients from the transformed negative control image. Statistical significance was tested with an unpaired sample t-test, showing a significant loss of correlation for VAMP7 and Stx17 when fusion is inhibited.

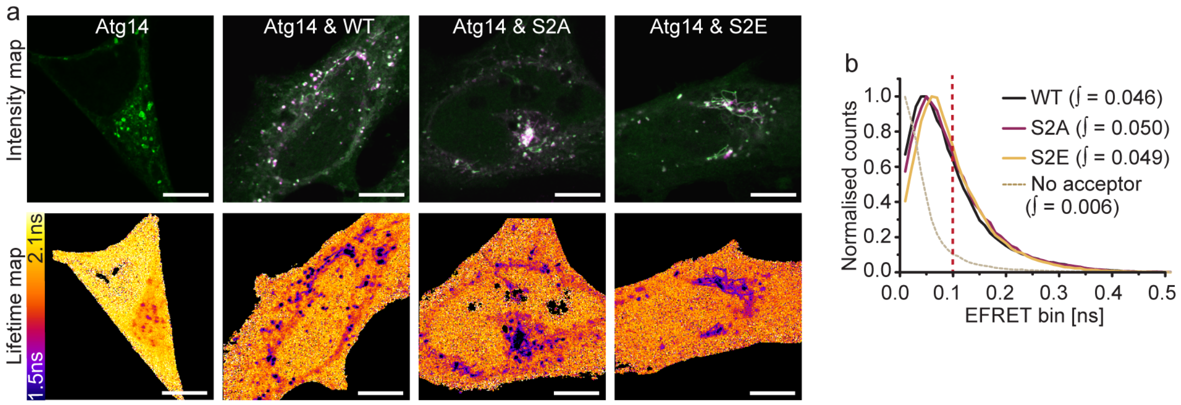

Supplementary figure 4 Atg14 interacts with wild-type Stx17 and its N-peptide mutants

(**a**) Intensity and fluorescence lifetime maps of rapamycin-treated HeLa cells expressing EGFP-Atg14 alone or alongside mCherry-fused Stx17[WT], Stx17[S2A] or Stx17[S2E] as indicated. (**b**) Single-pixel normalised FRET efficiency histograms accumulated from all fields for the dataset presented in (a). FRET efficiency integral values above 0.1 were tested for statistical significance using a one-tailed unpaired two-sample t-test [n=4]. Box-and-whisker plots represent the median (central line), 25th and 75th quartile (box) and 1st and 99th quartile (whiskers). Scale bars are 10 μm.

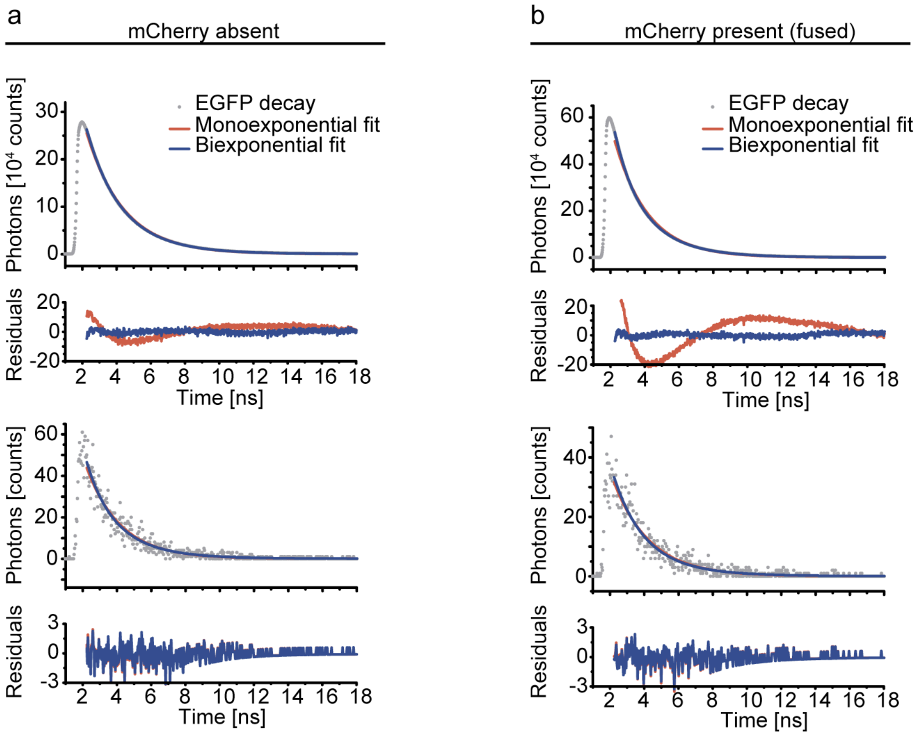

Supplementary figure 5 Representative EGFP fitted decays

(**a**) Whole cell (top) and single pixel (bottom) fluorescence decays for EGFP expressed alone in HeLa cells. Mono-exponential (red) and bi-exponential (blue) fits are shown with their corresponding weighted residuals below, indicating improved fit statistics with a bi-exponential equation. (**b**) As for (a) using data acquired from HeLa cells expressing EGFP fused to mCherry.
